# Achieving Ultrahigh Hardness in Electrodeposited Nanograined Ni-Based Binary Alloys

**DOI:** 10.3390/nano9040546

**Published:** 2019-04-04

**Authors:** Xiangui Zheng, Jian Hu, Jiongxian Li, Yinong Shi

**Affiliations:** 1 Shenyang National Laboratory for Materials Science, Institute of Metal Research, Chinese Academy of Sciences, Shenyang 110016, China; xgzheng@imr.ac.cn (X.Z.); jhu11s@alum.imr.ac.cn (J.H.); jiongxianli15s@imr.ac.cn (J.L.); 2 School of Materials Science and Engineering, East China Jiaotong University, Nanchang 330013, China

**Keywords:** electrodeposition, nanograined alloy, thermal stability, annealing hardening, hardness

## Abstract

Annealing hardening has recently been found in nanograined (ng) metals and alloys, which is ascribed to the promotion of grain boundary (GB) stability through GB relaxation and solute atom GB segregation. Annealing hardening is of great significance in extremely fine ng metals since it allows the hardness to keep increasing with a decreasing grain size which would otherwise be softened. Consequently, to synthesize extremely fine ng metals with a stable structure is crucial in achieving an ultrahigh hardness in ng metals. In the present work, direct current electrodeposition was employed to synthesize extremely fine ng Ni-Mo and Ni-P alloys with a grain size of down to a few nanometers. It is demonstrated that the grain size of the as-synthesized extremely fine ng Ni-Mo and Ni-P alloys can be as small as about 3 nm with a homogeneous structure and chemical composition. Grain size strongly depends upon the content of solute atoms (Mo and P). Most importantly, appropriate annealing induces significant hardening as high as 11 GPa in both ng Ni-Mo and Ni-P alloys, while the peak hardening temperature achieved in ng Ni-Mo is much higher than that in ng Ni-P. Electrodeposition is efficient in the synthesis of ultrahard bulk metals or coatings.

## 1. Introduction

To obtain ultrahard bulk materials or coatings has long been the goal of material science. Although grain boundary (GB) strengthening is applicable for most metallic materials, it fails when grains fall into an extremely fine regime when strength or hardness deviates from the conventional Hall-Petch relationship [1,2] and turns out to be softened [3,4,5]. It has been recently [6] reported that by employing appropriate annealing, softening can be avoided by stabilizing GBs through the GB relaxation and GB segregation of solute atoms in extremely fine nanograined (ng) metals. With a stabilized GB, the hardness of extremely fine ng metals is inversely proportional to the grain size, illustrating the possibility of designing ultrahard ng materials at an elevated temperature. Nevertheless, the synthesis of extremely fine ng metals is challenging. When the grain size is smaller than 10 nm, the volume fraction of GBs and triple junctions (TJs) gets very high, which provides a strong driving force for grain growth; grain coarsening occurs even at an ambient temperature [7]. Based on the concept of stabilizing the ng structure through alloying proposed by Weissmüller [8,9] and Kirchheim [10], it is expected to synthesize extremely fine ng materials in alloy systems.

Electrodeposition has been widely employed in the preparation of bulk pure metals, alloys or their coatings. So far, there have been over 300 kinds of binary alloys that can be electrodeposited [11]. As a “bottom-up” approach to preparing ng material, electrodeposition is undoubtedly one of the promising methods to synthesize extremely fine ng alloys. In comparison with “top-down” approaches, such as severe plastic deformation, the advantages of electrodeposition lie mainly in the readily obtainable homogeneous microstructure via controlling reactions happening on the solid/liquid interface by adjusting both chemical composition of electrolyte and operational parameters, especially for those alloys with a large difference in the melting point that are normally difficult to process through traditional metallurgy approaches.

It has been reported for electrodeposited single-phase binary alloys that the grain size is closely related to the alloy composition [12,13,14,15,16,17,18]; the higher the alloying content, the smaller the grain size. The composition of an alloy is determined not only by the bath composition but also by operational parameters like the current density, pH value, operating temperature, etc. [19,20,21,22]. The critical issue in the synthesis of single-phase ng alloys with an extremely fine grain size lies in the control of the alloying element content, i.e., introducing as much alloying element as possible to the ng structure on the one hand, while avoiding the formation of a second phase on the other hand. It is reported [20] that when the content of the alloying element exceeds a certain limit, the grain size will unexpectedly increase instead of decreasing with the presence of more alloying elements. 

In the present paper, the synthesis of extremely fine ng alloys by direct current electrodeposition will be explored in Ni-Mo and Ni-P alloy systems. Mo was selected as the alloying element based on the fact that Mo has a big atomic size mismatch with Ni. P, on the other hand, is highly immiscible in Ni at the equilibrium state. According to the criterion proposed by Koch et al. [23], both Mo and P are promising elements in stabilizing the ng structure. It is also considered that Mo and P have different segregation tendencies in Ni [24], which is believed to be critical in stabilizing the ng structure [25]. By comparing the grain size dependence of ng alloys on the Mo and P content and the subsequent annealing hardening phenomena, their effect in achieving ultrahigh hardness at an elevated temperature will be discussed.

## 2. Materials and Methods

Direct current electrodeposition was employed for the preparation of both ng Ni-Mo and Ni-P, where 99.99% nickel plates were used as the anode, and electro-polished copper plates were used as the cathode. Nickel-rich aqueous citrate-ammonia electrolytes were used for the Ni-Mo electrodeposition with Na_2_MoO_4_·2H_2_O to supply Mo (VI) ions, sodium citrate was used as the complexing agent, and sodium lauryl sulfate was used as the wetting agent. The electrolytes for the Ni-P preparation consisted of NiSO_4_·6H_2_O, NiCl_2_·6H_2_O, H_3_PO_4_ and H_3_PO_3_ without the addition of additives. The electrodeposition was carried out under a galvanostatic condition. In the Ni-Mo bath, a mixture of 2 g/L of saccharin and 0.15 g/L of 2-butyne-1,4-diol was introduced to reduce the grain size as well as the surface roughness. The pH value of the Ni-Mo bath was adjusted by ammonia. The electrolyte was stirred by a magnetic stirring bar at a constant rotating speed of 400 rpm and 800 rpm for the Ni-Mo and Ni-P electrodeposition, respectively. The thicknesses of all the deposited alloys were about 70 μm. Detailed bath compositions and deposition parameters are listed in Table 1.

Compositional examinations of each sample were carried out by energy dispersive spectrometer (EDS Oxford Instruments PLC, Abingdon, UK) in a Nova NanoSEM 430 operated at 15 kV. Structural characterization was conducted by X-ray diffraction (XRD) by using Cu-Kα radiation in a Rigaku D/Max 2400 operated at 100 mA and 40 kV. Transmission electron microscope (TEM) and high resolution transmission electron microscope (HRTEM) observations were performed on a Tecnai G^2^ F20 operated at 200 kV. The grain sizes of the as-deposited Ni-Mo and Ni-P alloys were determined by using both XRD and TEM dark field observations. To prepare the TEM specimens, free standing Ni-Mo and Ni-P samples were stripped from the Cu substrate, 3 mm discs were subsequently punched and mechanically polished to a thickness of about 30 μm, followed by twin-jet electropolishing in a 9:1 solution of alcohol and perchloric acid at 253 K and a voltage of 20 V. 

Atom probe tomography (APT) was employed to investigate the distribution of elements in the as-deposited Ni-Mo and Ni-P alloy samples. APT for the Ni-Mo experiments was performed on a Cameca local electrode atom probe LEAPTM 4000X Si at a specimen temperature of 20 K, under a pulsing UV laser with a wavelength of 355 nm and a laser energy of 40 pJ, with a pulse repetition rate of 200 kHz and an ion collection rate of 1% per pulse. The APT data reconstruction and quantitative analysis were carried out using the CAMECA IVAS version 3.6.8 software (CAMECA, Gennevilliers, France). For the detailed procedure, please refer to the SOM of [6]. APT for the Ni-P analysis was performed on a Cameca LEAP 3000 HR (CAMECA, Gennevilliers, France) operating at a base temperature of 70 K in voltage pulsing mode with a 15 pulse fraction and 200 kHz pulse frequency.

The as-deposited samples were annealed at different temperatures for 1 h, protected in an argon atmosphere. Microhardness tests were performed on a Qness Q10A + Micro-Hardness Tester with a load of 50 g and a holding time of 10 s. Each hardness value was averaged with at least 15 indentations.

## 3. Results

### 3.1. Synthesis

One critical issue in the synthesis of single-phase extremely fine ng alloy is to introduce as much solute atoms as possible to the alloy system without the formation of an amorphous phase or second phase. As summarized by Brenner [12], Ni-Mo electrodeposition follows an induced codeposition mechanism. The commonly used ammonia citrate aqueous solution was selected where sodium molybdate was used as a Mo (VI) source. With fixed operating parameters like the pH, current density, temperature, etc., the Mo content is controlled by altering the composition of sodium molybdate from 0.8 to 8 g/L. Although electroless depositions are normally used to prepare Ni-P, electrodeposition was employed in the current work to avoid the shortcomings of a high operating temperature and unreadily controlled deposition rate in electroless depositions. Ni-P electrodeposition is also classified as an induced codeposition. The present bath is a typical Brenner type solution [26] with phosphorous acid as the P supplier and phosphoric as the buffer. The concentration of phosphorous varied from 0.3 to 5.0 g/L to control the P content. Detailed bath compositions and operating conditions for the synthesis of ng Ni-Mo and Ni-P are listed in Table 1. 

It needs to be clarified that besides the grain size, a smooth shiny surface appearance is also considered as a prerequisite for ng Ni-Mo and Ni-P to provide a functional performance in property tests and practical applications [11]. For Ni-Mo deposits, the surface quality gets poorer with the increase of the Mo content in the alloy, which may come from the dramatically decreased current efficiency [11] and increased internal stress. The addition of a mixture of 2-butyne-1,4-diol and saccharin [16] greatly improves the surface appearance and reduces the surface roughness. However, Ni-P retains a fairly good-looking appearance regardless of the P content, and no additives were added in the ng Ni-P deposits; sodium dodecyl sulfate (SDS) was included just for the ease of hydrogen evolution.

**Figure 1:**
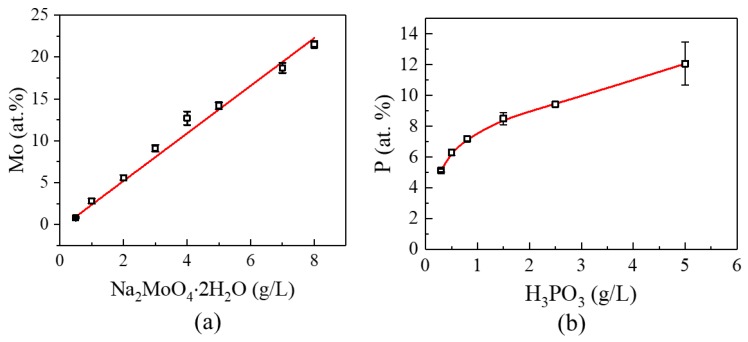
(**a**) Correlation between Mo content and concentration of sodium molybdate (Na_2_MoO_4_·H_2_O) (**b**) P content in Ni-P alloy vs. concentration of phosphorous acid (H_3_PO_3_).

EDS was carried out to detect the Mo and P content respectively on the Ni-Mo samples prepared from the solutions with different sodium molybdate concentration and Ni-P alloys from electrolytes with varying phosphorous acid addition. We found that the Mo content in the Ni-Mo deposits varies linearly with the concentration of sodium molybdate in the electrolyte when other components and operating parameters are fixed (Figure 1a). The atomic percentage of Mo is about 0.8 with the addition of 0.5 g/L sodium molybdate in the bath, and the value reached 21.5 when the sodium molybdate concentration increased to 8 g/L. Further increase of the sodium molybdate concentration could result in peeling off even with the simultaneous presence of saccharin and 2-butyne-1,4-diol. However, different from that of Ni-Mo, the atomic percentage of P in Ni-P alloys increased monotonically but nonlinearly with the concentration of phosphorous acid in the electrolyte, as shown in Figure 1b. The P content increased sharply when little phosphorous acid was added, and it reached 5 at.% when there was only 0.3 g phosphorous acid in the 1 L solution, while the value went to around 12 atomic percentage when the concentration of phosphorous acid increased to 5 g/L. A higher P content than 12 at.% leads to an amorphous structure. Brenner’s early work [26] showed a similar trend to our data but lower P contents, which probably came from the different operating parameters. 

### 3.2. Structural and Chemical Characterization

**Figure 2:**
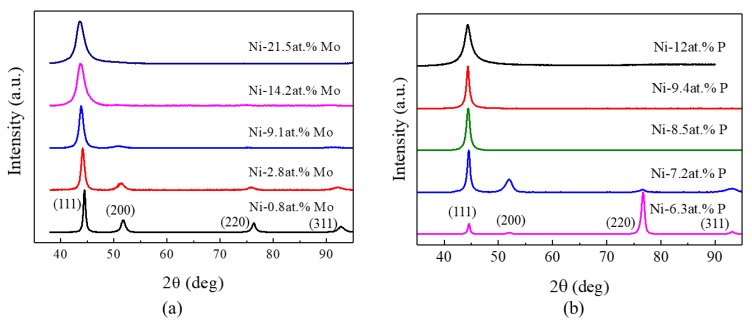
X-ray diffraction patterns of (**a**) ng Ni-Mo alloys with the atomic percentage of Mo varying from 0.8 to 21.5, and of (**b**) ng Ni-P with P from 6.3 to 12.

The microstructural characterization on the prepared Ni-Mo and Ni-P samples with different Mo and P contents was performed by XRD. Figure 2a presents the X-ray profiles of Ni-0.8 at.% Mo, Ni-2.8 at.% Mo, Ni-9.1 at.% Mo, Ni-14.2 at.% Mo [16] and Ni-21.5 at.% Mo. All the samples exhibit a strong (111) diffraction peak. Broadened diffraction peaks, as designated in the figure, are observed on Ni-0.8 at.% Mo, which correlates with the single-phase face centered cubic (fcc) structure. The peaks get broader, with more Mo atoms codeposited in the alloys. When the atomic percentage of Mo gets as high as 21.5, only the (111) diffraction peak is observable, showing a reduction of grain size with higher Mo contents. The grain sizes calculated from the peaks by using Scherrer equation are 16 nm, 11 nm, 8 nm, 4 nm and 2 nm, corresponding to ng the Ni-Mo samples with 0.8, 2.8, 9.1, 14.2 and 21.5 at.% Mo, respectively. The minimum average grain size of 2 nm was achieved in the Ni-21.5 at.% Mo sample.

The XRD patterns of the Ni-P deposits also exhibit a single-phase fcc structure (Figure 2b), and a similar trend to those of the ng Ni-Mo samples is observed, except that there appears a transition of a strong diffraction peak from (220) to (111), when the atomic percentage of P increases from 6.3 to 7.2 or higher. The grain size calculated from the diffraction peaks are 16, 13, 12, 12 and 4 nm, corresponding to the P content of 6.3, 7.2, 8.5, 9.4 and 12, respectively.

**Figure 3:**
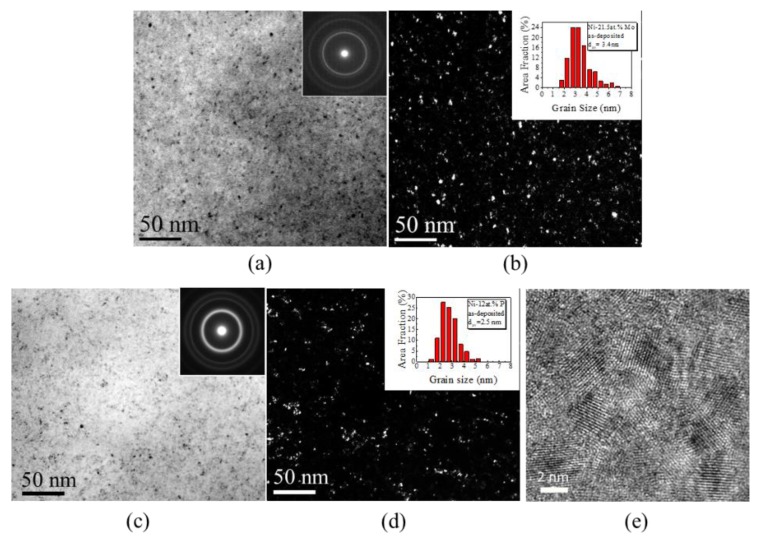
Typical (**a**,**c**) bright field (BF) and(**b**,**d**) dark field (DF) images of electrodeposited (**a**,**b**) ng Ni-21.5 at.% Mo and (**c**,**d**) Ni-12 at.% P. The insets of (**a**,**c**) and (**b**,**d**) are the corresponding selected area electron diffraction (SAED) patterns and grain size distributions, respectively. (**e**) A typical high resolution transmission electron microscope (HRTEM) image showing some tiny grains in ng Ni-12 at.% P.

TEM observations were carried out on these ng Ni-Mo and Ni-P samples. Figure 3a,b present typical bright field (BF) and dark field (DF) images of the ng Ni-21.5 at.% Mo sample, which show a homogeneous structure with many equiaxed nano-sized grains of a few nanometers. The inset of Figure 3a represents the selected area electron diffraction (SAED) rings, which indicate that the grains are randomly oriented. The inset of Figure 3b represents the histogram of the grain size distribution of the sample with an average grain size of 3.4 nm, a little bit higher than that calculated from XRD.

Figure 3c,d give the BF and DF images of Ni-12 at.% P. There appears no distinct difference between ng Ni-12 at.% P and Ni-21.5 at.% Mo in the BF and DF images, nor in the SAEDs (insets of Figure 3a,c). The statistic grain size distribution (the inset of Figure 3d) shows that the average grain size of Ni-P is as small as 2.5 nm, very close to that achieved in Ni-21.5 at.% Mo of 3.4 nm. 

The HRTEM images of Ni-12 at.% P (Figure 3e) confirmed that the as-deposited sample had a nanocrystalline structure with grain sizes of a few nanometers, as shown in Figure 3e. A similar ng structure was also observed in Ni-21.5 at.% Mo [6].

**Figure 4:**
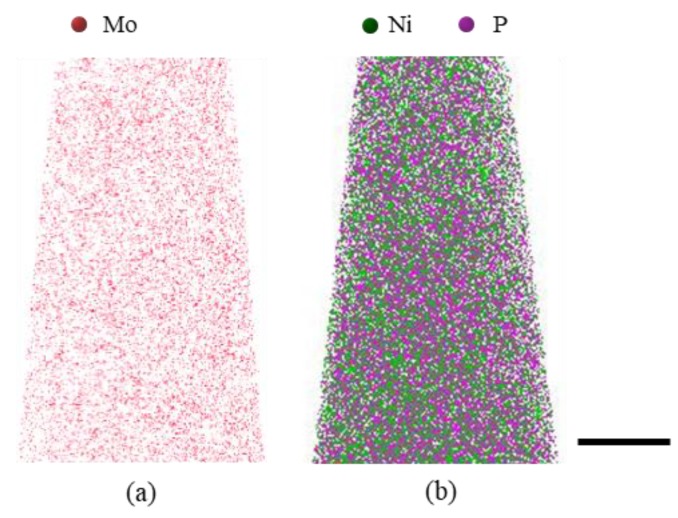
3D atomic probe tomography (APT) results of (**a**) as-deposited Ni-14.2 at.% Mo (red dots stand for Mo atoms) and (**b**) Ni-8.5 at.% P (green for Ni with purple P) exhibiting homogeneous distribution of both solute and solvent atoms (Ni, Mo and P). Scale bar in the figure is 20 nm.

The chemical compositions and their distributions of the ng Ni-Mo and Ni-P samples were characterized by using APT. The three dimensional (2D) reconstruction from the APT detections showed that solute atoms of Mo and P distribute homogeneously both in the as-deposited Ni-14.2 at.% Mo (Figure 4a) and in the Ni-8.5 at.% P samples (Figure 4b).

**Figure 5:**
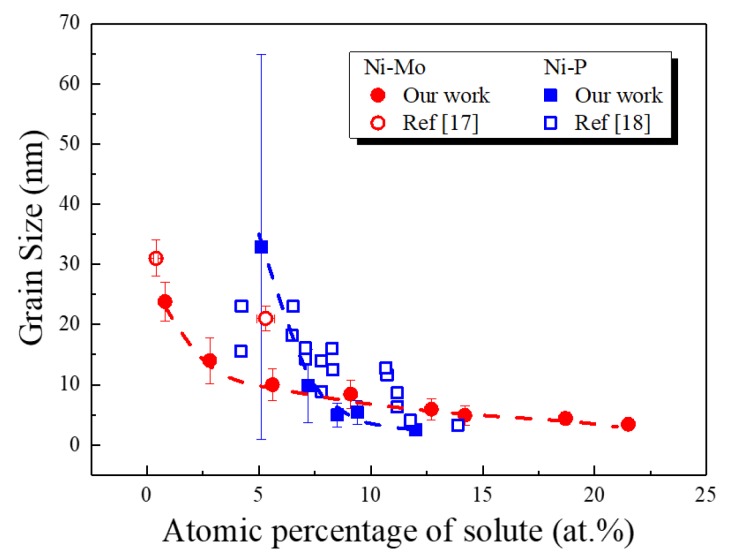
Grain size dependence of ng Ni-Mo alloys and Ni-P alloys on the atomic percentage of the solute atom (Mo and P), with the red line standing for Ni-Mo and the blue one representing Ni-P, respectively. The red circles stand for data of Ni-Mo, with blue squares representing those of Ni-P; the solid symbols are our results, with hollow ones being from references [17,18].

By plotting the grain size (averaged from DF images of the TEM observation) against the atomic percentage of solute Mo or P in the electrodeposited ng Ni-Mo and Ni-P samples (Figure 5), one can find that the grain size of either ng Ni-Mo or Ni-P decreases with the increase of the solute content. The average grain size of Ni-Mo is 24 nm when the atomic percentage of Mo is 0.8, and it gradually goes down to about 3 nm when there is 21.5 at.% Mo introduced into the alloy. While the grain size of Ni-P is 33 nm when there is 5 at.% P, the value drops quickly below 3 nm when the P content increases to only 12 at.%. The two lines intersect at a point where the content of the solute (Mo or P) is about 8% with the corresponding grain size around 7–8 nm. To get a minimum grain size of about 3 nm, more Mo atoms than P need to be included in the deposit. Recent work by Kapoor et al. in electrodeposited ng Ni-Mo alloys [17] and earlier work by Dietz [18] in electrodeposited Ni-P alloys are also included in Figure 5 for comparison. The two points of Kapoor et al. also showed a decrease in grain size from 31 nm to 21 nm when the Mo content was promoted from 0.4 to 5.3 at.%. In ng Ni-P, Dietz’s data scattered around ours, reaching a similar minimum grain size when the atomic percentage of P increased to 12–14.

### 3.3. Annealing Hardening

**Figure 6:**
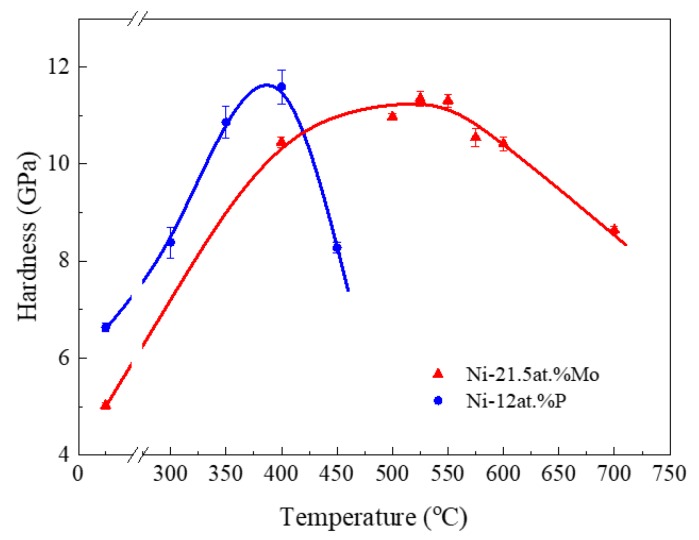
Variation of microhardness as a function of the annealing temperature with a duration of 1 h for ng Ni-21.5 at.% Mo (red line) and Ni-12 at.% P alloy (blue line).

We recently found that annealing at the appropriate temperature could introduce substantial hardening in extremely fine ng Ni-Mo alloys [6]. The maximum hardness increment is closely related to the initial grain size. The smaller the grain size, the higher the maximum hardness increment. Here, we compare the annealing hardening behavior of ng Ni-21.5 at.% Mo and Ni-12 at.% P. Microhardness measurements were conducted on these two ng samples after annealing them at different temperatures for 1 h. As depicted in Figure 6, the microhardness of as-deposited Ni-12.0 at.% P is 6.6 ± 0.1 GPa, and the value increases to 8.4 ± 0.3 GPa after the sample was annealed at 300 °C for 1 h. With the annealing temperature increasing further to 400 °C, the hardness reaches a maximum value of 11.6 ± 0.4 GPa. The microhardness of the annealed sample begins to drop when the annealing temperature gets higher than 400 °C. Similar to the annealing hardening curve of ng Ni-P, annealing also promoted the microhardness of Ni-21.5 at.% Mo from 5.0 ± 0.1 GPa (as-deposited) to the maximum of 11.4 ± 0.2 GPa (525 °C annealed), followed by a drop trend. However, the peak hardness temperature corresponding to the maximum microhardness of Ni-Mo is 125 °C higher than that of Ni-P (400 °C). That is to say, ng Ni-21.5 at.% Mo accomplished its maximum hardness at a higher temperature than the ng Ni-P alloy. 

**Figure 7:**
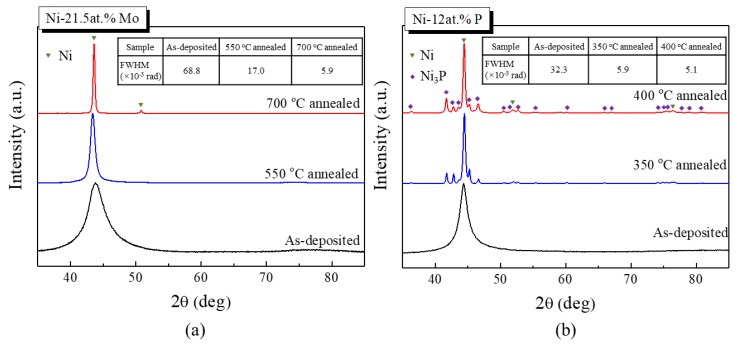
X-ray diffraction patterns of as-deposited and annealed (**a**) Ni-21.5 at.% Mo and (**b**) Ni-12 at.% P. The green nabla in the figures designate the diffraction peaks of Ni and the purple diamonds represent those of Ni_3_P.

To reveal the microstructure evolution during annealing, XRD detection was performed on the annealed Ni-21.5 at.% Mo and Ni-12 at.% P samples, as presented in Figure 7a,b, respectively. Compared with the XRD pattern of the as-deposited Ni-21.5 at.% Mo sample, the (111) diffraction peak narrowed when annealed at 550 °C. The TEM observation indicated that the corresponding grain size increased to about 15 nm. After being annealed at 700 °C for 1 h, more diffraction peaks of fcc appear in the diffraction pattern of ng Ni-Mo. In addition, the full-width at half-maximum (FWHM) value of the (111) diffraction peak (inset of Figure 7a) decreased tremendously from the initial 68.8 × 10^−3^ to 5.9 × 10^−3^ rad, exhibiting a significant grain growth corresponding to the much lower hardness of about 8 GPa, as shown in Figure 6. Although no diffraction peaks of a second phase were detected by XRD, precipitation at this annealing temperature cannot be excluded.

Different from ng Ni-Mo, observable diffraction peaks of Ni_3_P appeared when the annealing temperature was 350 °C. After annealing at 400 °C, the diffraction peaks of Ni_3_P got strengthened slightly, indicating the emergence of more Ni_3_P phase. In the meantime, the FWHM of Ni (111) dropped from 32.3 × 10^−3^ to 5.9 × 10^−3^ and further to 5.1 × 10^−3^ rad, representing a fast grain coarsening. The good temperature correlation between the precipitation of Ni_3_P and the maximum microhardness of ng Ni-P upon annealing clearly shows that precipitation strengthening is at least partly responsible for annealing hardening in extremely fine ng Ni-P alloys. Annealing hardening in electrodeposited ng Ni–P alloys was also reported by Chang [27], with lower phosphorus content.

## 4. Discussion

To synthesize extremely fine ng materials with a grain size down to a few nanometers in binary alloys, it is essential to select appropriate solute atoms that have a segregation tendency. According to Chookajorn [25], the competition of the enthalpy of segregation and the enthalpy of mixing between the solute (Mo and P in the current case) and solvent (Ni) determines the structural stability of an ng alloy. Koch et al. proposed that the stabilization of an ng alloy should take atomic mismatch and solubility into account [23]. The atomic radius of the solvent Ni atom is 1.246 Å, while that of Mo is 1.39 Å [28]. The atomic size mismatch between Ni and Mo is 11.6%, and this atomic mismatch introduced larger elastic mismatch energy with more Mo atoms inclusion, thus promoting structural stability and achieving progressively finer grain sizes (Figure 5). The continually shifting of the XRD (111) peak to lower angles with increasing Mo atoms exhibited in Figure 2a reflected this fact. On the other hand, although P has a comparable average atomic volume with Ni [29], which was also supported by the present result in Figure 2b with a fixed diffraction peak position irrespective of the P content, the bulk interaction energy with Ni is higher than that of Mo, similar to that reported between P and W [30]. Therefore, P has a stronger segregation tendency than Mo in Ni, in accordance with that calculated by Murdoch [24]. Although the minimum grain sizes that were achieved by electrodeposition in Ni-Mo and Ni-P alloys are both around 3 nm, the incorporated global solute content of Mo is higher than that of P (21.5 at.% Mo vs. 12 at.% P). The result in Figure 5 is in accordance with what Trelewicz and Schuh [30] proposed: lower segregation energies would generally change the equilibrium grain size-composition trends upward and exhibit a gentler decrease in the grain size with an increasing global solute content for nanostructure stabilization in binary systems. By incorporating an appropriate amount of solute atoms, extremely fine ng alloys have been successfully synthesized by direct current electrodeposition from aqueous solutions in Ni-Mo and Ni-P binary systems, indicating that electrodeposition is a powerful approach in the preparation of ng alloys with a relatively stable microstructure.

To achieve ultrahigh hardness in ng materials, structural stability is as crucial as grain size. By introducing stable nanotwins in cubic boron nitride, ultrahigh hardness twice as high as its coarse-grained counterparts was achieved [31]. By the generation of an ultra-stable nanolaminated structure in the surface layer of Ni, an ultrahigh hardness of about 6.4 GPa was reported [32]. Similarly, as exhibited in Figure 6, the microhardnesses of ng Ni-12 at.% P and ng Ni-21.5 at.% Mo were promoted tremendously by the enhanced GB stability after a low temperature annealing. Annealing hardening has been reported experimentally in ng pure metals [33,34,35] as well as in ng alloys [6,27,36], which was normally ascribed to the GB relaxation [37] and GB segregation of solute atoms. In extremely fine ng metals, we have found that appropriate annealing substantially promotes the GB stability which governs the deformation mechanism and the corresponding mechanical response in extremely fine ng Ni-Mo alloys [6]. The generation of partial dislocation is responsible for the very high annealing hardening in Ni-21.5 at.% Mo in Figure 6, as there was no significant grain growth at the temperature when the maximum hardness was obtained. In Ni-12 at.% P, the peak hardening temperature corresponded to the temperature where a significant amount of precipitates (Ni_3_P) precipitated, demonstrating that these precipitates contributed to the microhardness increment. In an early work [26] on an electrodeposited Ni-P alloy with 13–14% P showed that it reached its maximum hardness of about 9 GPa upon annealing at 400 °C, in accordance with our result. This temperature also correlates well with the temperature where an exothermic decomposition peak appeared in the thermal analysis of electrodeposited Ni-P with similar P content [38]. However, precipitation also resulted in the loss of the GB stability [39], as maintaining stability in extremely fine ng alloys requires the segregated species homogeneously decorating the grain boundary network rather than precipitating out in the form of secondary or impurity phases [40].

Owing to the early precipitation of Ni_3_P, the peak annealing temperature which correlated with the maximum microhardness of ng Ni-P is lower than that of ng Ni-Mo. That is to say, ng Ni-Mo achieves a higher thermal stability than ng Ni-P, although P is a stronger segregation solute than Mo. Therefore, in designing stable ng metallic materials with superior strength or hardness, not only the segregation tendency, but also the precipitation tendency, has to be taken into account.

## 5. Conclusions

Ng Ni-Mo and Ni-P alloys with an extremely fine grain size were successfully synthesized by electrodeposition. To get a grain size down to about 3 nm, fewer P atoms are needed in ng Ni-P than Mo atoms in ng Ni-Mo. Annealing induces significant hardening in both extremely fine ng Ni-Mo and Ni-P, leading to a similar hardness as high as 11 GPa in the two ng Ni binary alloys. The peak hardening temperature achieved in ng Ni-Mo is higher than that in ng Ni-P. The current work confirms the efficacy of electrodeposition, a “bottom-up” approach, in achieving ultrahigh hardness at elevated temperature in ng binary alloys.

## Figures and Tables

**Table 1:** Electrolyte composition and operating condition for the electrodeposition of ng Ni-Mo and Ni-P.

	Ni-Mo		Ni-P	
	NiSO_4_·6H_2_O	60 g/L	NiSO_4_·6H_2_O	150 g/L
	Na_3_C_6_H_5_O_7_·2H_2_O	80 g/L	NiCl_2_·6H_2_O	45 g/L
Composition	NaMoO_4_·2H_2_O	0.5–8.0 g/L	H_3_PO_4_	40 g/L
	Saccharin	2 g/L	H_3_PO_3_	0.3–5.0 g/L
	2-butyne-1,4-diol	0.15 g/L	SDS	0.2 g/L
pH	~9		~4	
Temperature (°C)	35		50	
Current density (mA/cm^2^)	30		50	
